# Lifetime Prevalence of Transient Loss of Consciousness in an Urban Russian Population

**DOI:** 10.5935/abc.20160056

**Published:** 2016-05

**Authors:** S. Gudkova, N. Cherepanova, D. Duplyakov, G. Golovina, S. Khokhlunov, E. Surkova, O. Rotar, A. Konradi, E. Shlyakhto

**Affiliations:** 1 Samara Regional Cardiology Dispensary, Samara - Russiam; 2 Samara State Medical University, Samara - Russiam; 3 Samara Medical Clinical Centre, Togliatti - Russiam; 4 Federal North-West Medical Research Centre, Saint-Petersburg - Russiam

**Keywords:** Unconsciousness / epidemiology, Syncope, Vasovagal, Cross-Sectional Studies, Urban Population

## Abstract

**Background:**

Most international studies on epidemiology of transient loss of consciousness (TLC) were performed many years ago. There are no data about the lifetime prevalence of TLC in Russia.

**Objective:**

To identify the lifetime prevalence and presumed mechanisms of TLC in an urban Russian population.

**Methods:**

1796 individuals (540 males [30.1%] and 1256 females [69.9%]) aged 20 to 69 years (mean age 45.8 ± 11.9 years) were randomly selected and interviewed within the framework of multicentre randomised observational trial.

**Results:**

The overall prevalence of TLC in the studied population was 23.3% (418/1796), with the highest proportion (28%) seen in 40-49 year age group. TLC was significantly more common in women than in men (27.5% vs 13.5%). The mean age of patients at the time of the first event was 16 (11; 23) years, with 333 (85%) individuals experiencing the first episode of TLC under 30 years. The average time after the first episode of TLC was 27 (12; 47) years. The following mechanisms of TLC were determined using the questionnaire: neurally-mediated syncope (56.5%), arrhythmogenic onset of syncope (6.0%), nonsyncopal origin of TLC (1.4%), single episode during lifetime (2.1%). Reasons for TLC remained unidentified in 34% cases. 27 persons (6.5%) reported a family history of sudden death, mainly patients with presumably arrhythmogenic origin (24%).

**Conclusion:**

Our findings suggest that the overall prevalence of TLC in individuals aged 20-69 years is high. The most common cause of TLC is neurally-mediated syncope. These data about the epidemiology can help to develop cost-effective management approaches to TLC.

## Introduction

Even after publication of Framingham study in 2002 we still have to say that data about epidemiology and prognosis of transient loss of consciousness (TLC) in the community are lacking.^1^ The prevalence of TLC, often described as a "blackout" or a "collapse", in population has a bimodal distribution with peaks in teenagers (13-15 years) and in the elderly (after 70 years). In the young, almost all cases of TLC have neurally-mediated origin, while in the elderly, cardiac causes and orthostatic hypotension predominate. However, the lifetime prevalence of TLC is difficult to obtain due to recollection bias of fainting episodes which occurred many years ago.^2,3^ Available evidence as well as limitations of multiple cohort and population-based studies on epidemiology of TLC are summarized in European Guidelines for the diagnosis and management of syncope and recent reviews.^4-6^

The objectives of this study were to identify the lifetime prevalence and presumed mechanisms of TLC in an urban Russian population.

## Methods

The data analysed in this paper were collected as part of a multicentre cross-sectional study of the epidemiology of Cardiovascular Disorders in Regions of the Russian Federation (ESSE-RF). Participants were from a random population sample of the residents of the city of Samara (1.1 million residents). Multi-stage random sampling was used to select people:^7^

-four outpatient clinics were randomly selected from a total of 17 clinics in Samara City;
-within these clinics, 10 residential catchment areas were randomly selected;
-within catchment areas, 50 households per area were randomly selected.

A survey was conducted by specially trained medical staff. Initially, people were invited to participate in the study by postcards and/or by phone calls. If we did not receive any response, than researchers went to the selected homes, explained the goals of the study, and invited them directly. The results were recorded using structured questionnaires and then entered into a database. Initially individuals from 25 to 64 years were recruited, while patients from 20 to 24 years of age and from 65 to 69 years of age were added later, using the same selection principles. Of the 2200 individuals approached, we were successful in recruiting 1796, resulting in a response rate 81.6%.

Twelve data collection modules used in ESSE-RF Trial were supplemented by the additional module specifically designed for the purposes of our study.^8^ The following self-reported information was collected in this module: 1) family history of sudden death (SD) related to cardiac pathology in first-degree relatives (parents and siblings) under the age of 45 years; 2) one or more episodes of having had a sensation of pulsation or movement in the chest; 3) a history of TLC: age at the time of the first event, as well as 14 questions that allowed to suspect neurally-mediated mechanism of TLC. This questionnaire (Table 1) was used in previous work to diagnose the neurally-mediated syncope (NMS) with a sensitivity of 95% and a specificity of 57%.^9^ In addition, information was obtained from medical records concerning doctor-diagnosed episodes of paroxysmal palpitations and their main characteristics, such as duration of the attack, type of heart rhythm, its association with TLC, suddenness of the onset and offset, ECG recording during the attack, and its interpretation. Blood pressure measurement, rest ECG and cholesterol levels were obtained for all recruited patients according to the protocol of ESSE-RF study.

**Table 1:** Questionnaire to diagnose the neurally-mediated origin of transient loss of consciousness^9^

**Questions**	**Points (if, yes)**
Do you have a faint without obvious reasons?	-3
Do you have a faint with head rotation?	-3
Have you ever had spells or faint lying in bed?	-2
Have you ever had spells or faint while walking?	-2
Do you experience aura (strange light, unpleasant smell, confusing thoughts) before a faint?	-2
Do you need more than 30 minutes to recover after a faint?	-1
Do you feel drowsiness during recovery period?	-1
Do you faint in hot places?	1
Have you ever had spells or faint during medical procedures?	1
Do you have blurred vision before a faint?	1
Do you feel warm before a faint?	1
Do you have warning symptoms more than 30 seconds before your faint?	2
Do you faint with prolonged standing?	2
Have bystander noted you to be pale during your faints?	3

Neurally-mediated syncope may be considered if the point score is ≥ 1

We defined TLC as an episode of spontaneous loss of consciousness not associated with brain injury, and followed by spontaneous recovery of consciousness regardless of the underlying mechanism. Syncope was defined as TLC related to temporary total brain hypoperfusion.^3^ The tentative mechanism of TLC was determined on the basis of criteria proposed by the European Society of Cardiology.^5^

Statistical analysis was performed using Statistica software 7.0. Data were presented as mean values and standard deviations (M ± s) in case of normal distribution, and as medians (Me) and 25 and 75 percentile values if the distribution was non-normal. Statistical analysis for normally distributed data was made using two-sided unpaired *t-* test for continuous variables and X^2^-test for categorical ones. Otherwise, Wilcoxon-Mann-Whitney test was used for comparison between groups. A two-tailed p value < 0.05 was considered statistically significant.

## Results

### General characteristics of the patients with TLC

**Figure 1:**
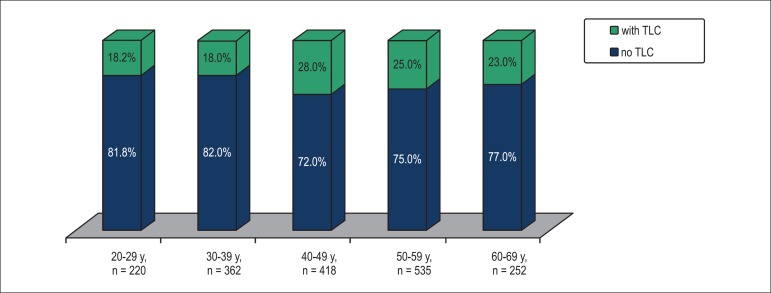
Lifetime prevalence of transient loss of consciousness (TLC) by age at survey.

This study enrolled 1796 individuals (mean age was 45.8 ± 11.9 years; 1256 women and 540 men), 418 of whom reported a history of TLC during lifetime, resulting in an overall lifetime prevalence of TLC of 23.3%, with the highest level of 28% in those aged 40-49 years (Figure 1). In men, TLC prevalence (13.5%; mean age 45.3 ± 11.2) was significantly lower than in women (27.5%; mean age 47.6 ± 11.2), p < 0.01.

The vast majority of interviewed individuals (79.9%) had no concomitant heart condition. One-fifth reported arterial hypertension, and 15 patients (3.5%) suffered from coronary artery disease (CAD). On ECG, premature ventricular complexes were recorded in 20 subjects (4.8%), atrial fibrillation in 5 subjects (1.2%), and WPW syndrome in 3 (0.7%). Other 23 patients (5.5%) reported recent episodes of arrhythmias on ECG, but did not know their exact type. Twenty-seven individuals (6.5%) reported SD among first degree relatives under age of 45 years.

**Figure 2:**
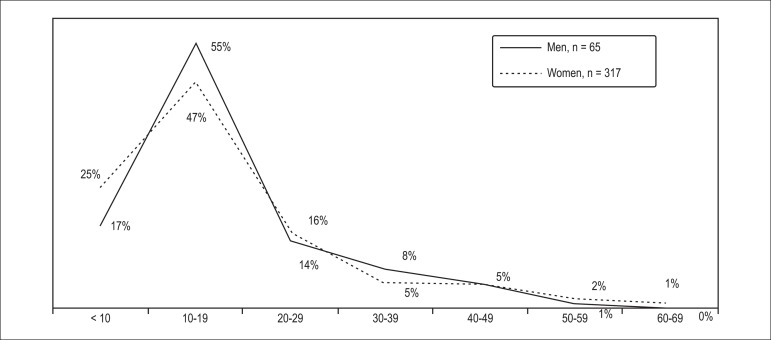
The age of patients at the first episode of transient loss of consciousness (years).

The mean age of patients at the time of the first TLC episode was 16 (11; 23) years, with 333 (85.0%) individuals (both men and women) experiencing their first episode of TLC before 30 years of age (Table 2). Almost half of the patients (55% men and 47% women) experienced the first TLC aged 10-19 years (Figure 2). The prevalence of the first episode of TLC in the population declined with age, being as low as 1% in women aged 60-69 years. The average time between first reported episode of TLC and the survey was 27 (12; 47) years.

**Table 2:** Clinical characteristics of patients with transient loss of consciousness (TLC)

	**NMS**	**Arrhythmogenic TLC**	**Nonsyncopal**	**Single episode**	**Unidentified reason**
# (%) of patients	236 (56.5%)	25 (6%)	6 (1.4%)	9 (2.1%)	142 (34%)
Score by questionnaire	2.33 ± 1.77	0.91 ± 0.05	-4.17 ± 1.72	0.38 ± 0.11	-1.69 ± 1.37
Mean age, years	46.5 ± 11.1	55.4 ± 7.9	52.2 ± 8.5	49.3 ± 9.6	46.5 ± 11.8
Male	18.6%	12%	0%	0%	14.8%
Mean age at the debut of TLC, years	15.6 ± 11.0	17.4 ± 9.9	19.3 ± 8.7	NA	16 (11;22)
The length of clinical history of TLC, years	26.8 ± 14.5	28.7 ± 12.1	32.0 ± 14.9	NA	25 (13;36)
Family history of sudden cardiac death	5.1%	24.0%*	0%	0%	6.3%

NMS: neurally-mediated syncope ; NA: not applicable;

* p < 0.01. TLC: transient loss of consciousness.

**Figure 3:**
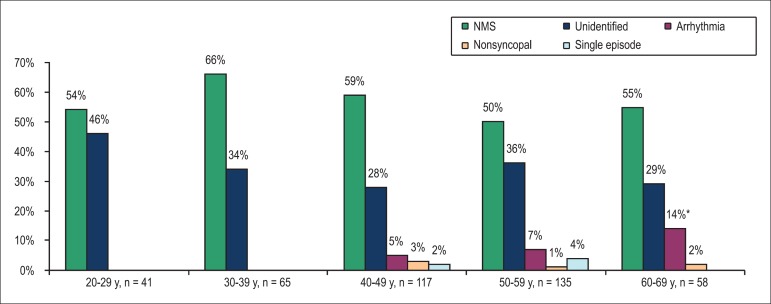
Prevalence of different classes of transient loss of consciousness by age (* p < 0.01). NMS: neurally-mediated syncope.

Based on the data obtained from the questionnaire, we classified the likely underlying mechanisms of first reported episode of TLC in the study population (Table 3). In all age groups, NMS predominated, being observed in 50% to 66% of individuals (Figure 3). TLC of presumably arrhythmogenic origin first appeared in patients > 40 years of age, and its frequency increased in older patients, reaching 14% in 60 69 year age category (p < 0.01). TLC due to nonsyncopal reasons or a single episode of TLC was also seen in older patients only (> 40 years). The prevalence of cases with unidentified reason varied from 46% in the youngest age group to 28%-36% in other age groups.

**Table 3:** Prevalence of presumed mechanisms of transient loss of consciousness (TLC)

**Presumed mechanisms**	**Prevalence**
NMS	56.5%
Arrhythmogenic onset of syncope	6.0%
Nonsyncopal origin of TLC	1.4%
Single episode of TLC	2.1%
Unidentified genesis of TLC	34.0%

NMS: Neurally-mediated syncope; TLC: transient loss of consciousness.

### Presumed NMS

Based on the data collected using the questionnaire, NMS as a tentative mechanism of TLC was identified in 236 (56.5%) individuals. Mean questionnaire score within this group was 2.33 ± 1.77.

One or more typical NMS triggers identified through the questionnaire included: syncope occurring in crowded and hot places, 172 (72.9%) individuals; syncope associated with prolonged time in standing position, 38 (16.1%) individuals; syncope associated with intravenous injections, medical procedures, 57 (24.2%) individuals; syncope associated with neck movement, 8 (3.4%) individuals; syncope associated with severe pain or emotional stress, 3 (1.3%) individuals.

Less than half of the individuals (106; 44.9%) with presumably NMS had overt and typical prodromal symptoms, mostly "blurred vision" in 101 (95.3%) patients, and feeling of warmth in 31 (29.2%) patients. Other patients (130; 55.1%) experienced little (100 patients; 42.4%) or no symptoms at all (30 patients; 12.7%).

In most cases, the recovery was quick, and consciousness restored rapidly. However, in 43 (18.2%) individuals, complete recovery took 30 minutes or more, and 15 (6.4%) individuals experienced drowsiness after TLC.

### Presumed arrhythmogenic origin of TLC

Twenty-five (6%) individuals reported palpitation sensations before TLC. Thirteen (52%) patients described their episodes of palpitation as having sudden onset and a regular pulse, whereas 9 (36%) patients experienced irregular heartbeats during palpitation. Only 16 patients (64%) sought medical help, the following cardiovascular disorders being diagnosed in 7: CAD (n = 3); arterial hypertension (n = 2); atrioventricular reciprocating tachycardia (n = 1); and atrial fibrillation (n = 1).

Using the questionnaire, we failed to identify risk factors potentially associated with life-threatening ventricular arrhythmias. Only 2 (8%) individuals experienced TLC while walking, and none of them complained of loss of consciousness in horizontal position.

In 11 (44%) individuals from this group, symptoms of arrhythmia were combined with typical NMS triggers. Episodes associated with crowded and hot environments were observed in 10 individuals, syncopes associated with prolonged time in standing position in 4 individuals, and syncopes associated with intravenous injections in 2 individuals. Six of those 11 patients experienced blurred vision and/or had warm sensation at the debut of TLC. Eight patients required more than 30 minutes to recover, and 5 individuals reported drowsiness after an episode. Thus, in a proportion of patients in this group, NMS may also have contributed to the development of TLC.

### Presumed nonsyncopal origin of TLC

In 6 (1.4%) women, loss of consciousness was not associated with any obvious cardiovascular symptom, but they experienced visual or auditory hallucinations during prodromal period. None of them had any comorbidity. In 2 women, hallucinations were combined with impaired vision during prodromal period and one "hot flash", and collapse was sudden in 3 of them. Recovering phase lasted more than 30 minutes in 2 women, and 4 of them slept after the episode suggesting epilepsy as a contributing factor.

### Single episode of TLC

Nine (2.1%) women reported only one episode of TLC during lifetime. These occurred during pregnancy (n = 5), were associated with specific medical conditions (pneumonia, hepatitis, toxic shock, one each), or stress related (take-off of an aircraft - 1 patient). They earned 0.38 ± 0.11 points by questionnaire.

### Unidentified reason for TLC

Absence of any reported triggers and development of TLC "for no obvious reason" was the basis for including such individuals in the group with unidentified genesis of TLC. This group consisted of 142 (34%) individuals, and almost 78% of them (110 individuals) had no symptom of any cardiovascular disease. Hypertension had been diagnosed in 31 (21.8%) patients previously, CAD in 8 patients, and atrial fibrillation in 2 patients.

Sixty-five (45%) patients with TLC of unknown origin did not have any prodromal symptoms. "Blurred vision" during prodromal period was observed in 62 (43%) individuals, feeling of warmth in 17 (12%). Syncope with "sudden" fall was reported by 42 (29%) patients. Rapid and full recovery was seen in the vast majority of cases; however, in 21 (15%) patients, full recovery took over 30 minutes, and some of them (12 patients) slept for a while after TLC.

### Sudden cardiac death

Twenty-seven individuals (6.5%) reported family history of SD. The highest prevalence of SD in close relatives (n = 6, 24%) was recorded in patients with presumed arrhythmogenic origin of TLC, which was significantly higher compared to patients with presumably NMS (5.1%) and unidentified reasons (6.3%). There was no SD among relatives of persons from the nonsyncopal and single episode groups.

## Discussion

Despite numerous publications, data about the incidence and prevalence of TLC in general population and different clinical settings are lacking, because some of them were published decades ago.^2,3,6,10-12^ To our knowledge, this is the first report of lifetime prevalence of TLC in urban Russian population. The overall lifetime prevalence of one or more episodes of TLC in our population was 23%, with the highest prevalence of 28% observed in individuals aged 40-49 years. In women, TLC prevalence was twice as high compared to men (27.5% and 13.5%, respectively, p < 0.01).

The first episode of TLC is known to commonly occur in people aged 10-30 years, with overall frequency peaking at 13-15 years and after 70 years of age.^12-15^ These data were confirmed in our study: the mean age at the time of first TLC in Samara city population was 16 (11; 23) years, with 85% of all TLC developing in patients under 30 years of age. However, contrary to the Framingham study results,^1^ in our population the risk of TLC decreased with age. This fact may be partially explained by the absence of individuals older than 70 years in our study, since the second peak of TLC occurrence in the Framingham study was observed at the age of 70 years, for both men and women.

The prevalence of TLC varies with age and its epidemiological characteristics could be significantly affected by diagnostic criteria and methods used in a particular study. The value of standardized questionnaires for screening has been demonstrated in multiple studies.^16-20^ In our study, we also used specialized questionnaire, but no further diagnostic procedures, and medical examination was conducted according to the protocol of ESSE-RF study.

The most commonly presumed cause of TLC in our population was NMS, which predominated in all age and gender groups. Therefore, our results were generally in agreement with the previously reported TLC scenarios, NMS being the most common cause of TLC, while cardiogenic syncope is usually less frequent, mostly having an arrhythmic origin. In approximately one-third of cases, the syncope genesis remains unidentified.^1,5,21-25^

Information about the prevalence of various types of TLC is of utmost importance for clinical practice. Careful medical examination allows confirming the neurally-mediated mechanism in most patients with presumed NMS.^5^ Tilt-table test, carotid sinus massage, and implantation of event recorders may be recommended in approximately half of patients with suspected neurally-mediated TLC without typical symptoms in prodromal period. Importantly, in 15-30% of patients with NMS, the differential diagnosis may be complicated due to symptoms overlapping other types of TLC.^19,26-28^

In patients with suspected arrhythmic syncope, further diagnostic tests should be performed to exclude overt heart disease, mainly due to a high prevalence (24%) of SD among first-degree relatives. This is especially true for patients with syncope onset during physical exertion and short medical history of TLC. However, for patients having typical NMS triggers and prodromal symptoms, it is also important to examine the autonomous nervous system function, because, in some of those episodes, palpitation may be a sign of NMS, which is difficult to differentiate only on the basis of medical history.

Detailed neurological examination, including video-EEG monitoring, is recommended for patients with suspected epilepsy. At the same time, 3 out of 6 patients with nonsyncopal TLC had a long history of falls, "hot flash" as a prodromal symptom, paleness of the skin during loss of consciousness and a short recovery period. We therefore recommend that all diagnostic tests recommended for patients with NMS should be considered in this group.

In patients with unidentified origin of TLC, examination should start with an autonomous nervous system function assessment, due to a potentially high (40%) prevalence of NMS with atypical clinical presentations in this group.^29,30^ Heart diseases and arrhythmias have to be excluded as well, as they often constitute the main risk factors for SD in this category of patients.^1,5,31^

### Limitations

Our study has several limitations. First, despite a lot of methods used in clinical practice, there is no gold standard in syncope evaluation. We collected self-reported information and data from medical records, accompanied by ECG and blood pressure measurement, which are currently considered enough by European Society of Cardiology guidelines^5^ for the initial evaluation of the vast majority of patients with syncope.

Second, syncope with different underlying mechanisms might have common predisposing factors and prodromal symptoms. Thus, it can be sometimes challenging to distinguish them. However, NMS is considered the most common cause of TLC in population. We used a specialized questionnaire with proven accuracy to diagnose NMS in our study.

Third, the results obtained in our study may not be applicable to patients with TLC admitted to tertiary centers, where TLC prevalence and mechanisms will be different.

## Conclusion

Our findings suggest that the lifetime prevalence of TLC in individuals aged 20-69 years is high. The most common cause of TLC is NMS. History of SD in close relatives was recorded in 24% of patients with presumably arrhythmogenic TLC origin. These epidemiological data can help to develop cost-effective management approaches to TLC.
